# Supplementary material for: Electron transport of WS2 transistors in a hexagonal boron nitride dielectric environment
Source: Sci Rep. 2014 May 15;4:4967. doi: 10.1038/srep04967 (PMC4021321; doi:10.1038/srep04967)
Supplement: Supplementary Information — Electron transport of WS2 transistors in a hexagonal boron nitride dielectric environment [file srep04967-s1.pdf]

Electron transport of WS_2 transistors in a hexagonal boron nitride dielectric environment: Supporting Information

F. Withers[1], T. H. Bointon[1] D. C. Hudson[1] M. F. Craciun[1] and S. Russo[1]

[1] *Centre for Graphene Science, CEMPS, University of Exeter, Exeter, EX4 4QL, UK*

1 Device fabrication details.

1.1 Materials

Synthetic WS_2 was purchased from lowerfriction.com. The specified particle size was 0.6 μm, although this only represents the mean particle size and large particles are present making it possible to obtain WS_2 flakes of lateral sizes of several microns.

1.2 Transfer technique for atomically thin flakes

The fabrication of WS_2 transistor in a BN dielectric environment is carried out following the procedures described in [1]. Figure S1 shows some micrograph images of these transistors. The transfer technique can be summarized as follows. We use a polymer double layer, water soluble conducting polymer AquaSave© and PMMA ($\approx$ 400 nm), and the flakes are cleaved onto the PMMA. The flake PMMA membrane is produced by placing a tape window over the flake and dissolving away the AquaSave© sacrificial layer. This free membrane can then be directly transferred onto any surface with micrometer accuracy by utilizing a micro manipulator stage of a conventional mask aligner system.

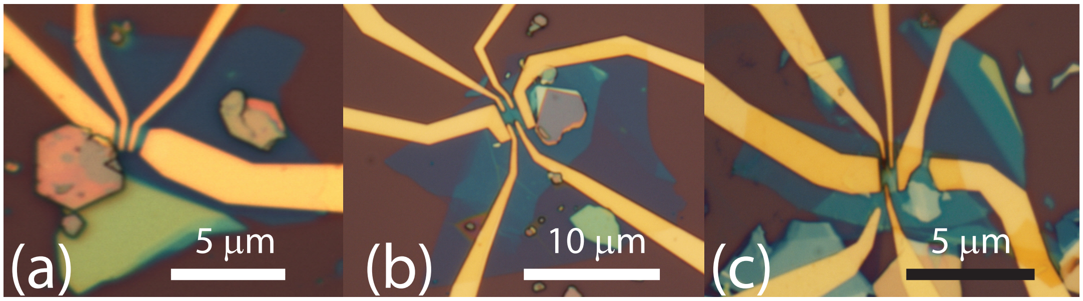

S1: (a) Optical image of a 4 L WS_2 flake transferred onto a BN substrate. (b,c) fully encapsulated devices, where a second BN flake has been transferred to fully seal the device.

2 High Bias treatment for enhanced electronic performance.

The electrical device performance of WS_2 transistors was improved by high bias sweeping of the source drain bias in high vacuum or inert helium environment. This process was effective only for the devices which were not encapsulated in BN. Figure S2 shows the current *versus* voltage characteristics of a representative WS_2 transistor. The observed improvement of the electrical properties can be summarized as follows. Initially the off-state of the transistor persists to several tens of volts. Above a certain source-drain bias threshold the device abruptly begins to conduct and in the next voltage bias sweep a much lower bias threshold is consistently observed. If the device is subsequently exposed to air for several hours a large source-drain bias threshold in the I-V is observed again. In this case a low source-drain bias threshold can be restored by repeating the aforementioned procedure. These observations suggest the presence of an insulating barrier at the interface between the metal contacts and WS_2.

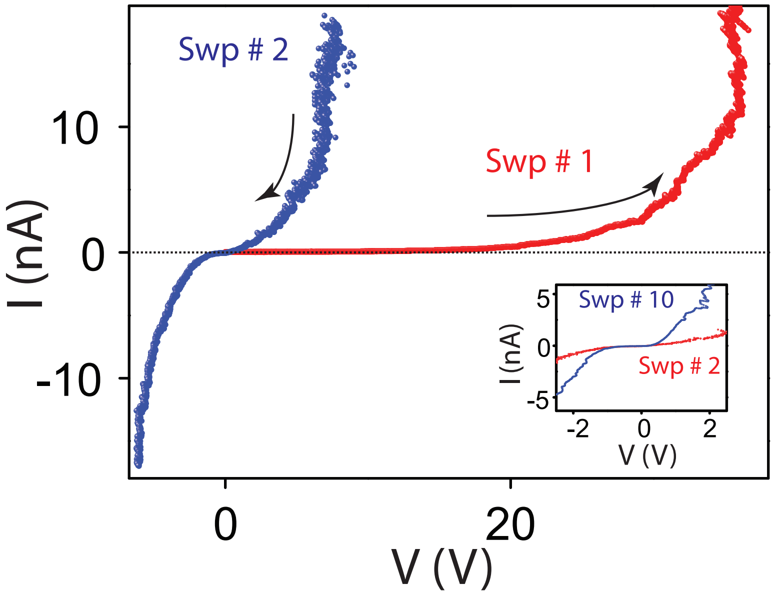

S2: I-V characteristics for a WS_2 transistor on silicon oxide substrate, the red curve is the initial sweep and the blue curve is the reverse sweep. (inset : red curve is again swp 2 and blue is the tenth subsequent sweep.)

We attempted to anneal the device shown in the main text for a 24 Hr period. The low temperature transport for the same device after such treatment is shown in Figure S3. It can be seen that the conductance peaks occur in a more ordered pattern possibly indicating a bunching or a narrowing of the impurity bands.

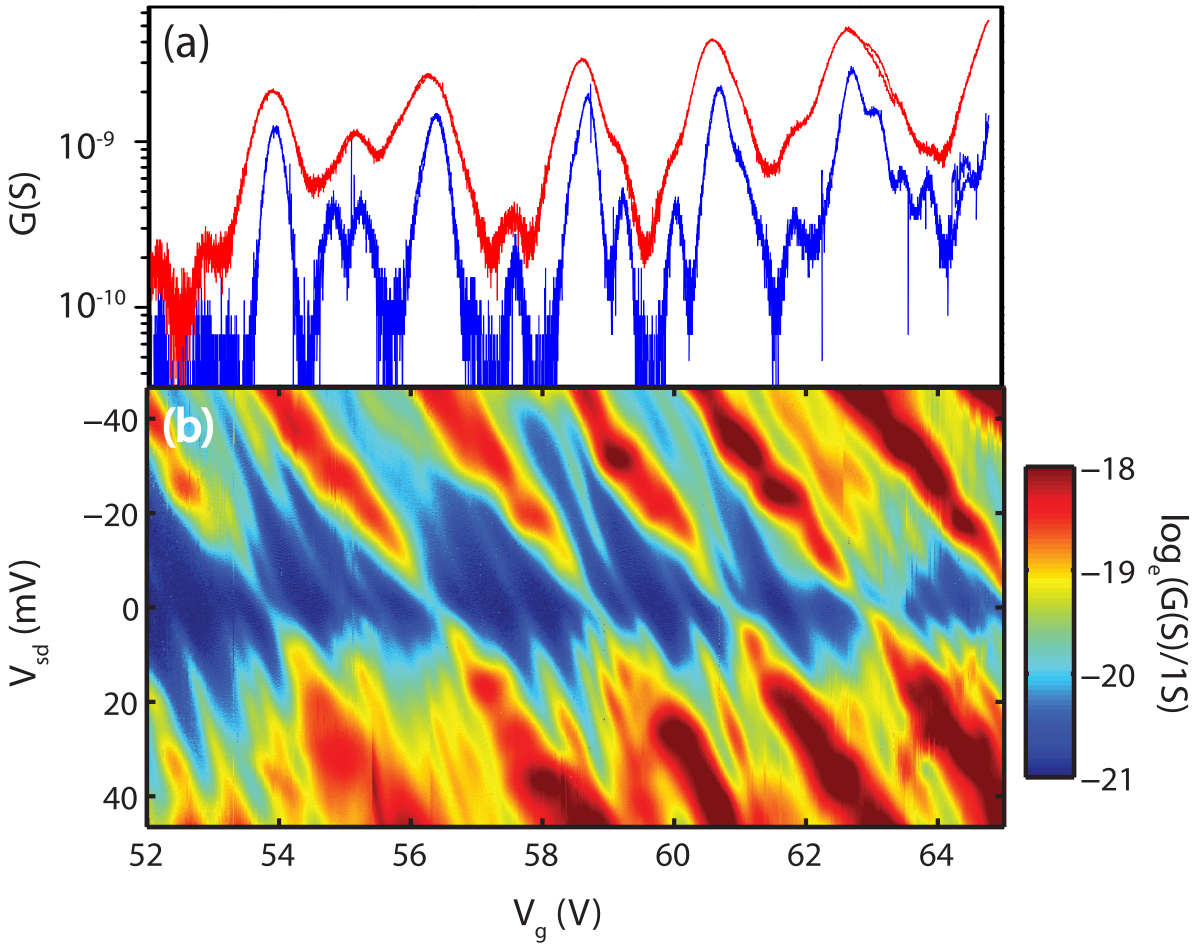

S3: (a) zero bias conductance vs gate voltage after 24 hrs annealing cycle for T = 4.2 K (blue) and T = 7 K (red) (b) The stability diagram for the device at T = 4.2 K.

In summary we show that high bias can effectively 'break' a dielectric barrier at the Cr/ WS_2 interface and that sustained high bias treatment significantly improves the ability to inject carriers into WS_2.

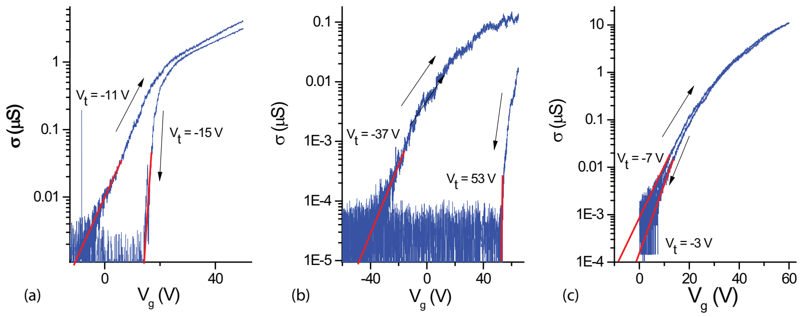

S4: The plots show the conductivity *vs.* gate voltage and the value of threshold voltage (V_t) for (a) a four-layer WS_2 on SiO_2, (b) single-layer WS_2 on SiO_2 and (c) four-layer WS_2 on hBN

Figure S4 shows the extrapolated values of threshold voltage from the graphs of conductivity as a function of gate voltage for the representative devices discussed in the main manuscript.

3 Hopping conduction of WS_2 flakes on Silicon oxide substrates.

Figure S5 shows the typical electron transport properties for a 4 L WS_2 flake on a silicon oxide substrate. It is seen that the transport behaviour is similar to that of WS_2 flakes on BN substrates indicating that defects in the crystal or on the surface of the crystal are responsible for localisation of electrons leading to hopping conduction through impurity states.

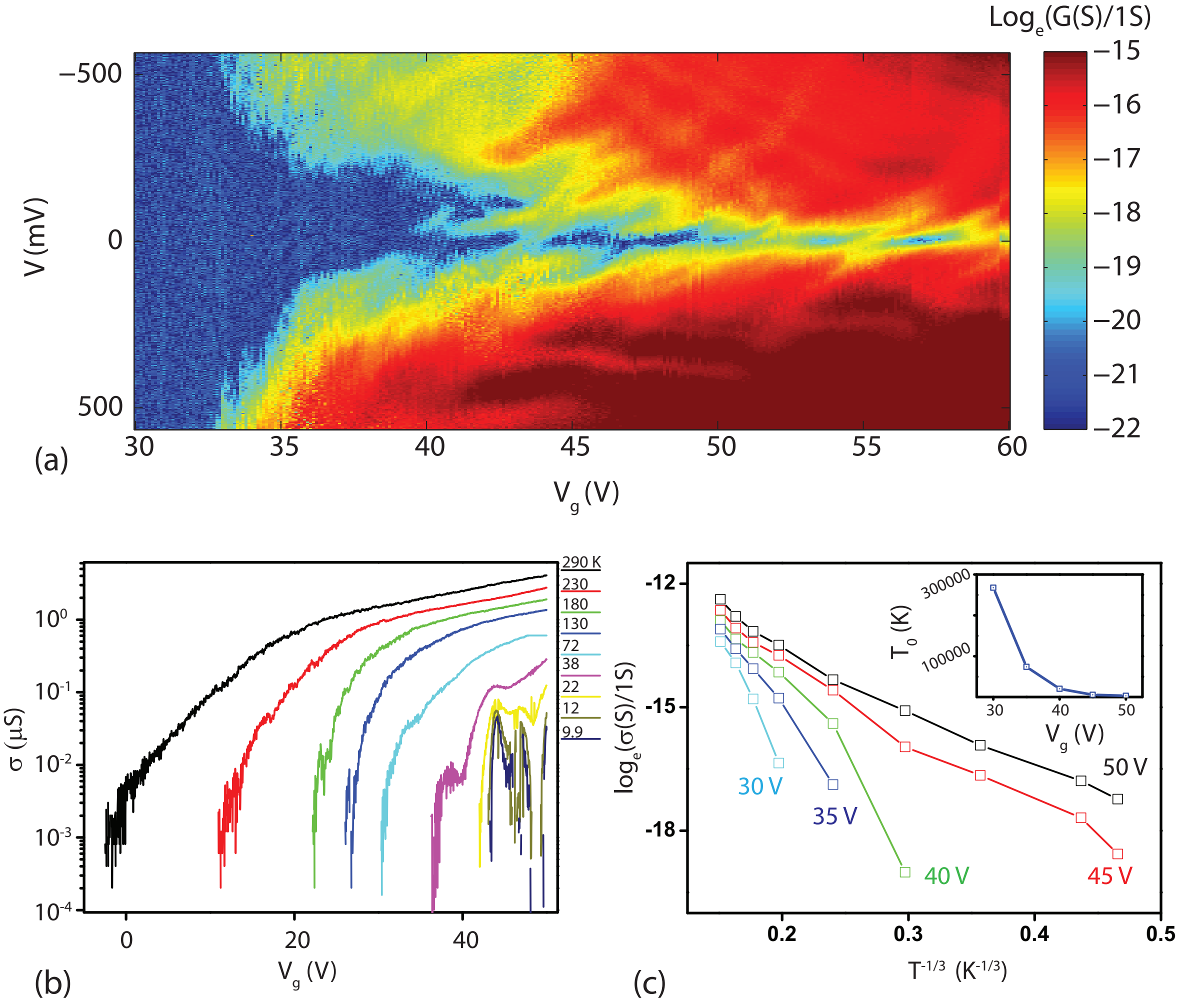

S5: (a) Bias and gate voltage dependence of the conductance once again at lower temperatures oscillations in the conduction at high gate voltage can be seen similar to the flakes on BN substrates. Temperature dependence of the conductivity again indicates 2D hopping behaviour.

4 Comparison of natural WS_2 and synthetic WS_2 crystals.

High quality graphene flakes are often derived from high quality graphite crystals with large atomic planes, such crystals are only obtainable through mining. By similar logic we attempted to study the properties of flakes exfoliated from natural tungstenite in the hope of being able to achieve larger flakes with better electronic properties. However, tungstenite is a rather rare earth mineral [2] and natural samples can only be obtained on the mm scale. We obtained flakes of WS_2 by microcleaving a 0.5 mm tungstenite crystal and we measured the Raman EDX and electrical transport properties of these natural flakes.

Figure S6(a) shows the elemental analysis [3] for synthetic and natural samples of WS_2 normalized to the lowest energy W peak. In the case of the synthetic material no alien impurity elements can be detected and the atomic ratio composition is found to be W:S 33 % :66 %. However in the natural crystal we see the presence of Mo, confirmed by Energy-dispersive X-ray spectroscopy see Figure S5 (a) with atomic ratio Mo:W:S 12.16 % :22.33 %:65.5 %.

The presence of Molybdenum severely modifies the phonon modes of the material as can be seen in figure S6 (b).

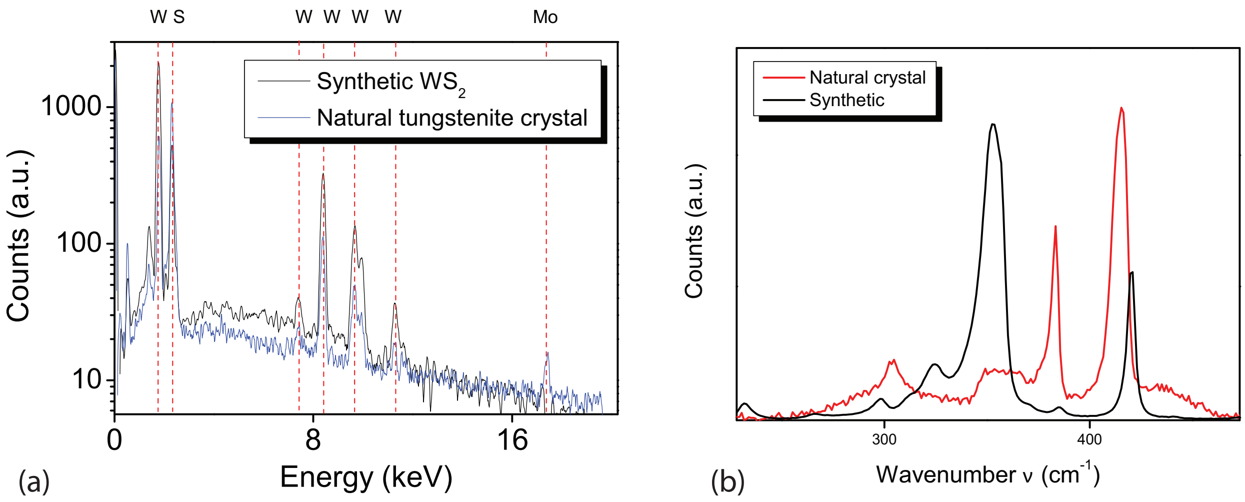

S6: (a) EDX spectra of a natural tungstenite sample blue data and of synthetically produced WS_2 black data, note the presence of Molybdenum in the case of the natural crystal. (b) Raman spectrum of the natural crystal of tungstenite (red data) and of synthetic material (black curve)

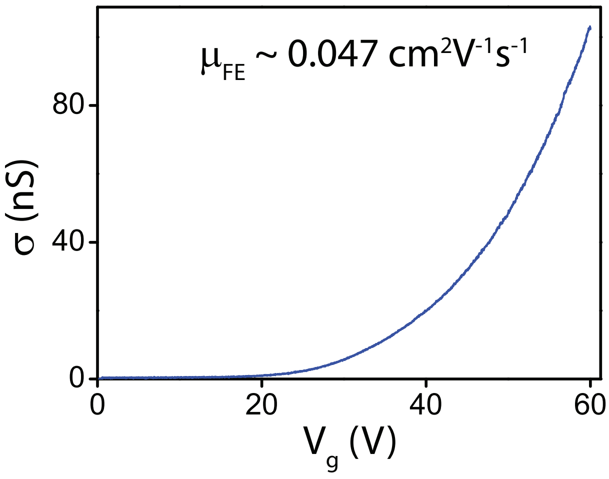

S7: Gate dependence of a few layer natural sample of tungstenite on a BN substrate. The device shows the lowest electronic quality of all the studied architectures.

References

[1] Dean, C. R et al., *Nat. Nano* **5**, 722 (2010).

[2] Klein, C and Cornelius S. H. Jr., Manual of Mineralogy, Wiley, 20th ed, 1986.

[3] Goldstein, J. et al., Scanning Electron Microscopy and X-ray Microanalysis, Kluwer Academic/Plenum Publishers, 2003.
